# Advancing global medical education in otolaryngology through hands-on skills training and simulation-based learning

**DOI:** 10.7189/jogh.11.03111

**Published:** 2021-10-23

**Authors:** Angela Cao, Jeremy Feintuch, Joshua Feintuch, Luan Tran, Brent Senior, Christina J Yang

**Affiliations:** 1 Department of Otorhinolaryngology-Head and Neck Surgery, Albert Einstein School of Medicine/Montefiore Medical Center, Bronx, New York, New York, USA; 2 Department of Otolaryngology-Head and Neck Surgery, Pham Ngoc Thach University of Medicine, Ho Chi Minh City, Vietnam; 3 Ear, Nose, and Throat Hospital of Ho Chi Minh City, Ho Chi Minh City, Vietnam; 4 Department of Otolaryngology-Head and Neck Surgery, University of North Carolina School of Medicine, Chapel Hill, North Carolina, USA

Global health is moving toward a focus on building strong and sustainable health systems in developing countries [1]. For global surgery, this means moving beyond short-term mission trips that solely deliver care and rather, towards education – teaching techniques, guiding post-operative care, and building surgical capacity of the local health care community. Global surgery collaborations should support surgical workforce development, education, and training of local surgeons in a manner that allows a bidirectional exchange of knowledge and resources [2], thereby developing the local capacity for sustainable surgical care.

In response to this call, one aspect of global surgery has shifted to in-country training of local surgeons [3]. Some global surgery groups have only recently started to formally incorporate these goals in their trips overseas. Fuller et al [3] employed a surgical curriculum in Ecuador focused on short lecture didactics, live surgery participation, and a surgical simulator to teach skills on various aspects of rhinoseptoplasty surgery. Vyas et al [4] report a curriculum based on virtual augmented reality to remotely proctor and guide surgical training of cleft-lip surgery for plastic surgeons in Peru. And Parham et al [5] designed a virtual reality surgical simulator to help novice surgeons perform radical abdominal hysterectomy surgery procedure in Zambia. We applaud these worthwhile endeavors, but also advocate the need for low-cost, easily assembled surgical simulators, especially in resource poor areas.

Surgical simulation has become an integral educational tool in United States surgical training programs. It has been widely established as a valuable and effective means of training surgeons prior to entering the operating room [6]. The American Academy of Otolaryngology-Head and Neck Surgery Foundation established the Otolaryngology Surgical Simulation Task Force in 2011, recognizing the importance of this tool in training surgical trainees [7]. The global surgical community recognizes its importance too and parts of the developing world are yearning for these tools. A recent review by Hasan et al [8] from Aga Khan University in Pakistan describes the need of simulation in surgical education in developing countries. While low- and middle-income countries (LMIC) institutions may be restricted to an apprenticeship model of learning surgery in a “see one, do one” manner due to either financial or resource constraints, we successfully introduced a low-cost simulation task trainer to develop sinus surgery skills that participants can directly learn and practice with hand-on training outside of the operating room.

**Figure:**
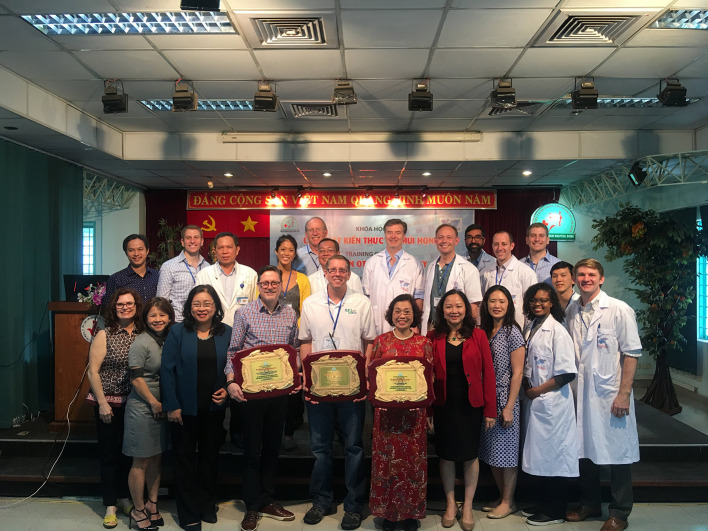
Photo: From the collection of Angela Cao, used with permission.

In recent years, our otolaryngology departments at Albert Einstein College of Medicine and the University of North Carolina at Chapel Hill have participated with Resource Exchange International (REI) Vietnam, a non-profit organization that has been working in Vietnam for over 25 years to build medical and health care capacity. These trips have been supported, in part, by the Vietnam Ministry of Health and led by American otolaryngologists since 1992, focusing on collaborations with medical universities with otolaryngology training programs in Hanoi and Ho Chi Minh City (HCMC). These institutions provide undergraduate and postgraduate training for the entire country. In collaboration with the Vietnamese Ministry of Health and Vietnamese physicians, our years of exchange work to accomplish the organization’s mission – to “build people to build a nation.”

During one of our recent REI Vietnam trips, we traveled to HCMC with a team of 20 volunteers, made up of American otolaryngology attendings, residents, and medical students. Our visit began with a 2-day course called “Update in Otolaryngology,” hosted by Pham Ngoc Thach University of Medicine and ENT Hospital of HCMC with lectures given by our group. This course is an annual highlight and attended by around 200 health care professionals from throughout the region. Through years of established partnership with our Vietnamese otolaryngology colleagues, an educational gap in endoscopic sinus surgery was identified among trainees with regard to dexterity and technical handling of instrumentation that could be addressed through ENT simulation-based training.

**Figure 1:**
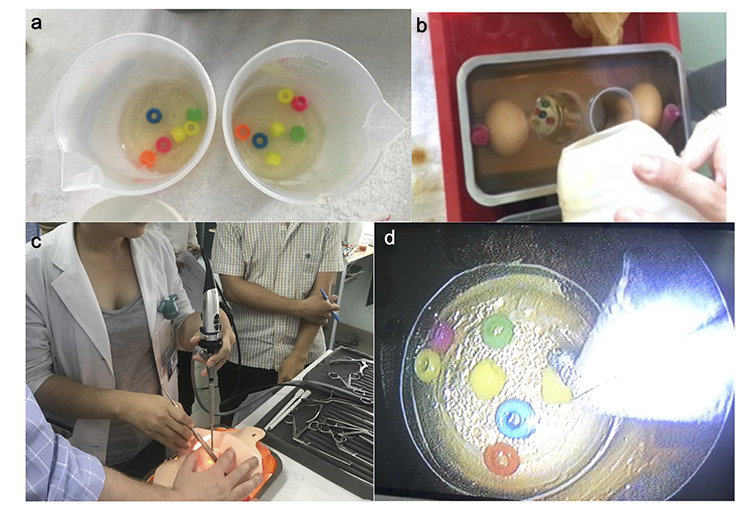
Assembly of the gelatin-based sinus surgery simulator. **Panel A.** Small discs of beads suspended in gelatin are prepared that were transferred into the simulator for the bead removal task. **Panel B.** Gelatin-based sinus surgery task trainer cooling in a baking pan. Eggs were suspended in gelatin to represent the maxillary sinus. Licorice candies were used laterally to position the eggs while the gelatin was cooling. **Panel C.** Sinus trask trainer with cardiopulmonary resuscitation (CPR) face mask in place. **Panel D.** Endoscopic view of bead removal task.

For our teaching objectives, we brought a task trainer to simulate endoscopic sinus surgery using a gelatin-based model established by Malekzadeh et al [9]. Developed to teach junior otolaryngology residents basic endoscopy and sinus surgery skills, we replicated this model with 11 Vietnamese otolaryngology residents and 1 Vietnamese otolaryngology attending. For less than US$5 dollars, we easily constructed and reproduced the task trainer on-site purchasing gelatin, beads, eggs, and licorice candy at local street markets in HCMC (**Figure 1**). The task trainer was used to simulate endoscopic sinus surgery tasks including uncinectomy, maxillary antrostomy, suture removal, and endoscopic injections. Tasks were completed using the model, a 0-degree endoscope, video tower, and standard sinus instruments.

After performing sinus endoscopy and the described tasks, participants provided feedback by completing an 18-question evaluation of the sinus trainer. This included questions used by Steehler et al [6] in assessing the validity of the sinus surgery task trainer developed by Malekzadeh [9]. A series of statements translated into both English and Vietnamese asked participants to rate the sinus model on its ability to develop general endoscopy skills and its ability to teach specific tasks and instrumentation using a 5-point Likert scale (strongly disagree to strongly agree). Results are shown in **Table 1**.

**Table 1:** Participant survey with 5-point Likert-type scale response

	Percentage responding (No.)					
**Statements and Likert-type scale responses**	**Strongly Disagree (Hoàn toàn không đồng ý**	**Disagree (Không đồng ý)**	**Neutral (Không ý kiến)**	**Agree (Đồng ý)**	**Strongly Agree (Hoàn toàn đồng ý)**	**Mean ± SD**
This model helps to develop camera skills needed for FESS (Mô hình này giúp phát triển kỹ năng sử dụng camera cần thiết cho phẫu thuật nội soi chức năng xoang)	0	0	0	25 (3)	**75 (9)**	4.8 ± 0.45
This model helps to develop hand-eye coordination needed for FESS (Mô hình này giúp phát triển khả năng phối hợp mắt - tay cần thiết cho phẫu thuật nội soi chức năng xoang)	0	0	0	17 (2)	**83 (10)**	4.8 ± 0.39
This model helps to develop skills needed for nasal endoscopy (Mô hình này giúp phát triển kỹ năng cần thiết cho nội soi mũi)	0	0	0	33 (4)	**58 (8)**	4.7 ± 0.49
This model helps to develop skills needed for frontoethmoid recess exploration (Mô hình này giúp phát triển kỹ năng cần thiết để khảo sát ngách sàng trán)	0	8 (1)	**42 (5)**	17 (2)	33 (4)	3.8 ± 1.06
The frontoethmoid recess task is a valuable training exercise (Bài tập về ngách sàng trán là huấn luyện có giá trị)	0	0	17 (2)	**58 (7)**	33 (3)	4.1 ± 0.69
This model helps to develop fundamental use of straight and 45-degree Blakesley (Mô hình này giúp phát triển kỹ năng sử dụng cơ bản kềm Blakesley thẳng và Blakesley 45 độ)	0	0	0	33 (4)	**67 (8)**	4.7 ± 0.49
The string removal task is a a valuable training exercise (Bài tập lấy bỏ các vòng xâu là huấn luyện có giá trị)	0	0	0	42 (5)	**58 (7)**	4.6 ± 0.51
This model helps to develop basic injection fundamentals needed for FESS (Mô hình này giúp phát triển kỹ năng chích tê cơ bản cần thiết cho phẫu thuật nội soi chức năng xoang)	0	0	0	25 (3)	**75 (9)**	4.8 ± 0.45
The basic injection task is a valuable training exercise (Bài tập chích tê cơ bản là huấn luyện có giá trị)	0	0	0	33 (4)	**67 (8)**	4.7 ± 0.49
This model helps develop dexterity, accuracy, and precision with sinus instruments (bead task) (Mô hình này giúp phát triển độ khéo léo, chính xác khi sử dụng dụng cụ phẫu thuật xoang (bài tập với hạt)	0	0	0	25 (3)	**75 (9)**	4.8 ± 0.45
The bead removal task is a valuable training exercise (Bài tập lấy bỏ hạt là huấn luyện có giá trị)	0	0	0	33 (4)	**67 (8)**	4.7 ± 0.49
This model helps to develop fundamentals involved in maxillary antrostomy (Mô hình này giúp phát triển kỹ năng cơ bản trong phẫu thuật mở lỗ thông xoang hàm)	0	0	17 (2)	33 (4)	**50 (6)**	4.3 ± 0.78
The maxillary antrostomy task is a valuable training exercise (Bài tập mở lỗ thông xoang hàm là huấn luyện có giá trị)	0	0	8 (1)	42 (5)	**50 (6)**	4.4 ± 0.67
Use of this model will increase resident competency when used to train residents prior to their first FESS (Tập luyện trước với mô hình này làm tăng khả năng của bác sĩ nội trú trước khi thực hiện lần phẫu thuật nội soi chức năng xoang đầu tiên)	0	0	0	25 (3)	**75 (9)**	4.8 ± 0.45
This model is an adequate training model for future surgeons (Mô hình này là hình thức luyện tập thích hợp cho các phẫu thuật viên tương lai)	0	0	0	17 (3)	**75 (9)**	4.8 ± 0.45
I would be interested in using this model to train residents (Tôi sẽ quan tâm sử dụng mô hình này để huấn luyện cho các bác sĩ nội trú)	0	0	0	0	**100 (12)**	5.0 ± 0.00
This model correlates with the essential skills needed for FESS (Mô hình này có tương quan với những kỹ năng cần thiết cho phẫu thuật nội soi chức năng xoang)	0	0	0	17 (3)	**75 (9)**	4.8 ± 0.45
This model is able to mimic actual sinus anatomy (high fidelity model) (Mô hình này giống với giải phẫu thật mũi xoang (độ tin cậy cao)	0	0	33 (4)	**42 (6)**	17 (2)	3.8 ± 0.72

SD – standard deviation, FESS - functional endoscopic sinus surgery

*Each Likert scale response is based on a 5-point scale response, strongly agree, agree, neutral, disagree, and strongly disagree corresponding with values 5, 4, 3, 2, 1 respectively. Responses are presented as a percentage. Mode response appears in bold for each statement. The last column represents the mean ± standard deviation based on the Likert scale value, with maximum possible score 5. The survey was administered to participants with statements in both English and Vietnamese.

The overall response to the sinus surgery task trainer was immensely positive. Ninety two percent of participants showed strong interest in using the task trainer to train residents. 100% agreed that the task trainer showed promise to simulating essential skills needed for functional endoscopic sinus surgery (FESS). 92% felt it was an adequate training model. And 92% felt it would increase resident competency. These percentages are comparable to the same favorable scores reported by Steehler et al [6] and Malekzadeh et al [9] in their validity studies for US trainees.

With our preliminary positive response from trainees in HCMC, we believe that surgical simulation stands not only to directly teach and transfer knowledge to otolaryngologists in training, but also answers the call for surgical capacity building in LMICs. There was immense enthusiasm for the surgical simulator by the surgical trainees who participated in the simulation. In fact, for many of the otolaryngology interns, it was their first time holding a sinus endoscope. During our multiple trips to Vietnam, we observed that the apprenticeship system in the operating room is not as robust as it may be in the United States. Some surgeries are broadcast to a classroom of trainees rather than having trainees in the operating room themselves. This may be even far more limited in more remote LMICs.

While it is not our intention to impose a Western educational system onto our Vietnamese colleagues, we believe that exposing and imparting various educational models such as surgical simulation will only open opportunities, especially with hands-on applications in surgical training. This aligns with global surgery’s renewed mission for capacity building and knowledge transfer. The path to establishing trusting and respectful relationships to embark on collaborations like these takes time, listening, and compassion. Local collaboration and participation are critical [10]. For these authors, we found it fruitful establishing relationships with strong government-affiliated tertiary care hospital that have cooperation with surgeons and trainees invested in education. By sharing our knowledge and furthering education with each other, we can continue to expand capacity of the field of otolaryngology globally.
